# Insomnia and cardiorespiratory fitness in a middle-aged population: the SCAPIS pilot study

**DOI:** 10.1007/s11325-018-1765-9

**Published:** 2018-12-13

**Authors:** Ding Zou, Heini Wennman, Örjan Ekblom, Ludger Grote, Daniel Arvidsson, Anders Blomberg, Kjell Torén, Göran Bergström, Mats Börjesson, Jan Hedner

**Affiliations:** 1 Center for Sleep and Vigilance Disorders, Department of Internal Medicine and Clinical Nutrition, Sahlgrenska Academy, University of Gothenburg, Medicinaregatan 8b, Box 421, SE-40530 Gothenburg, Sweden; 2 Department of Public Health Solutions, National Institute for Health and Welfare, Helsinki, Finland; 3 Åstrand Laboratory of Work Physiology, The Swedish School of Sport and Health Sciences, Stockholm, Sweden; 4 Center for Health and Performance, Department of Food and Nutrition, and Sport Science, Institute of Neuroscience and Physiology, University of Gothenburg, Gothenburg, Sweden; 5 Department of Public Health and Clinical Medicine, Division of Medicine/Respiratory Medicine, Umeå University, Umeå, Sweden; 6 Section of Occupational and Environmental Medicine, Sahlgrenska Academy, University of Gothenburg, Gothenburg, Sweden; 7 Department of Molecular and Clinical Medicine, Institute of Medicine, Sahlgrenska Academy, University of Gothenburg, Gothenburg, Sweden; 8 Sahlgrenska University Hospital, Gothenburg, Sweden

**Keywords:** Cardiovascular disease, Gender, Insomnia, Maximal oxygen consumption, Physical activity, Population-based cohort

## Abstract

**Background:**

The relationship between insomnia and cardiorespiratory fitness (CRF), a well-established risk factor for cardiovascular disease, has not been extensively studied. We aimed to assess the independent association between insomnia and CRF in a population-based cohort of subjects aged 50 to 64 years.

**Methods:**

Subjects participating in the Swedish CArdioPulmonary bioImaging Study (SCAPIS) pilot cohort (*n* = 603, men 47.9%) underwent a submaximal cycle ergometer test for estimation of maximal oxygen consumption (VO_2_max). Data on physical activity and sedentary time were collected via waist-worn accelerometers. An insomnia severity index score ≥ 10 was used to define insomnia.

**Results:**

Insomnia was identified in 31.8% of the population. The VO_2_max was significantly lower in insomnia subjects compared with the non-insomnia group (31.2 ± 6.3 vs. 32.4 ± 6.5 ml* kg^−1^ *min^−1^, *p* = 0.028). There was no difference in objectively assessed physical activity or time spent sedentary between the groups. In a multivariate generalized linear model adjusting for confounders, an independent association between insomnia status and lower VO_2_max was found in men, but not in women (*β* = − 1.15 [95% CI − 2.23–− 0.06] and − 0.09 [− 1.09–0.92], *p* = 0.038 and 0.866, respectively).

**Conclusions:**

We found a modest, but significant, association between insomnia and lower CRF in middle-aged men, but not in women. Our results suggest that insomnia may link to cardiovascular disease via reduced CRF. Insomnia may require a specific focus in the context of health campaigns addressing CRF.

**Electronic supplementary material:**

The online version of this article (10.1007/s11325-018-1765-9) contains supplementary material, which is available to authorized users.

## Background

Insomnia is defined as problems with sleep initiation, consolidation, duration, or quality, despite an opportunity to sleep enough coupled with daytime impairment. Insomnia and insomnia-related symptoms are highly prevalent in adult populations, ranging from 5 to 30%, being more common among women than men and increasing with age [1]. During the past decades, the prevalence of insomnia symptoms has grown both in the USA and Europe [2–4] and mounting evidence shows that insomnia is highly comorbid with psychiatric disorders, e.g., depression [1]. Emerging data also suggest that insomnia constitutes a risk factor for cardiometabolic diseases [5, 6]. Several mechanisms have been proposed to link insomnia and cardiometabolic disease including endocrine dysregulation, inflammation, autonomic imbalance, and endothelial dysfunction [6, 7]. It is also suggested that subjects with insomnia symptoms engage in more adverse health behaviors such as smoking and alcohol consumption and are less physically active [2, 8].

Cardiorespiratory fitness (CRF) describes the capacity of the circulatory system to provide working muscles with oxygen during heavy working loads and is often expressed in terms of measured or estimated maximal oxygen consumption (VO_2_max) level [9]. Higher levels of CRF have been shown to associate with numerous health benefits including reduced burden of anxiety and depression [10]. Conversely, low levels of CRF predict increased cardiometabolic disease risk and all-cause mortality [11].

Studies addressing the association between insomnia and CRF are sparse. In a large community-based sample, there was a moderate inverse association between self-reported insomnia symptoms and CRF, assessed by peak oxygen uptake [12]. The total score on the Insomnia Severity Index (ISI) questionnaire was inversely associated with self-perceived fitness in a cohort of students [13]. Therefore, in the current study, we aimed to investigate the association between insomnia classified by ISI and CRF assessed by a submaximal exercise test in a population-based sample of middle-aged, urban Swedish men and women. We hypothesized that insomnia is associated with decreased VO_2_max, independently of objectively measured physical activity level.

## Material and methods

### Overview of the study cohort

The Swedish CArdioPulmonary bioImage Study (SCAPIS) is an ongoing national multicenter study with the focus on identification and investigation of cardiometabolic disease and related mechanisms in a cohort of 30,000 men and women, aged 50 to 64 years [14]. In 2012, a comprehensive pilot trial was conducted at the Sahlgrenska University Hospital in Gothenburg, Sweden, to examine the feasibility and financial and ethical consequences of SCAPIS. In detail, 2243 subjects were randomly selected from the Swedish population register stratified for low and high socioeconomic status (SES) area and 1111 agreed to participate. Participants underwent a 2-day protocol including imaging and functional tests of the heart, lungs, and metabolism as well as extensive health questionnaires. In addition, participants were asked to perform a submaximal fitness test and to wear an accelerometer during 7 days to objectively record daily physical activity. All participants gave their written informed consent and SCAPIS has been approved as a multicenter study by the ethics committee at Umeå University (Dnr 2010-228-31M). The protocol for the current study was approved by the regional Ethics Committee of Gothenburg (Dnr 638-16).

### Insomnia and sleep duration

The Insomnia Severity Index questionnaire was used to classify insomnia [15]. The ISI consists of 7 questions assessing the nature, severity, and impact of insomnia (total score ranges from 0 to 28). A cutoff of ISI score ≥ 10 was applied for insomnia diagnosis [16]. Subjective sleep duration was assessed by the question: “How many hours do you usually sleep during 24-hours” with response alternatives: “4 hours or less; 5 hours; 6 hours; 7 hours; 8 hours; 9 hours; 10 hours or more,” further divided into a categorical variable “subjective sleep duration < 6 hours” yes or no.

### Cardiorespiratory fitness and physical activity

Participants were invited to participate in a submaximal cycle ergometer exercise test for the assessment of VO_2_max. The test followed the Ekblom-Bak cycle ergometer protocol for adults [17] and included 4 min cycling (Monark ergometer 828E, Monark Exercise AB, Vansbro, Sweden) at two different workloads, respectively. The first workload was standard and low work rate (~ 30 W) and the second a higher, individually chosen work rate aiming for a perceived exertion of approximately 14 on the Borg scale [18]. The mean heart rate (Polar Electro Oy, Kempele, Finland) during the last minute at the low and high workload was recorded respectively. VO_2_max was estimated based on the difference in workload and heart rate between the two workloads using validated gender-specific equations [19]. Subjects with diagnosed heart condition, taking beta-adrenergic blockers, having pain in hips, back, or knees, with obesity or perceived inability to perform the test were excluded from the test.

Objective measurement of physical activity in the SCAPIS pilot trial has been described in detail previously [20]. Briefly, participants were instructed to wear the triaxial accelerometers (ActiGraph GT3X and GT3X+, Actigraph, LCC, Pensacola, FL, USA) on the right hip for seven consecutive days during waking hours but not when in water-based activities. Sampling frequency was set at 30 Hz and data were downloaded using the ActiLife software (v. 6.10.1, Actigrah LCC, Pensacola, FL, USA) as 60-s epochs with low frequency extension filtering. Participants with at least 600 min of accelerometer wear time for at least 4 days were accepted to be included in analyses. The vector magnitude counts per minute (cpm) were used to classify time spent sedentary (SED: 0–199 cpm) and moderate to vigorous intensity physical activity (MVPA: ≥ 2690 cpm) [21, 22]. Data for SED and MVPA are shown as mean daily percentage over the measurement period.

### Information on lifestyle, living conditions, and comorbidities

Weight (kg), height (m), and waist circumference (cm) were measured prior to the fitness test. Body mass index (BMI) was calculated as weight divided by square height (kg/m^2^). Self-reported smoking habit was classified as never smoker, occasional smoker, former smoker, and current active smoker. Alcohol consumption was assessed using the Alcohol Use Disorders Identification Test consumption (AUDIT-c) questionnaire where a total score of 5 or higher in men and 4 or higher in women was classified as risk drinking [23].

University education (yes; no) was used to describe the subjects’ education level. Participants were asked if they currently are engaged in an income-related job or not.

Self-reported chronic disease was identified for subjects who reported one or more of the following: diagnosed diabetes, cancer, or COPD, previous bypass surgery or hospitalization because of myocardial infarction/stroke. The question “Have you ever had clinically diagnosed sleep apnea (yes/no)?” was used to identify subjects with comorbid sleep apnea. Information about depression symptoms was based on the question: “During the past 12 months, have you experienced a period of two or more than two weeks when you have felt sad, downhearted or depressed? (yes/no).”

### Statistical analysis

Statistical analyses were performed using SPSS 25.0 (IBM, Armonk, NY, USA). Data are shown as mean (SD) or median [interquartile range IQR]. Distribution of the variables was analyzed using Kolmogorov-Smirnov test. Chi-square tests were used to compare categorical variables. Normally distributed variables were compared using independent sample *t* test and Mann-Whitney test was applied for non-normally distributed data comparison. Effect size of the independent *t* test was determined by Cohen’s d. The association between insomnia and CRF was analyzed using generalized linear models, adjusting for age, gender, BMI, waist circumference, SES, university education, income-related job, smoking, risky drinking, chronic disease, sleep apnea, depression symptoms, sleep duration < 6 h, percentage of MVPA, and percentage of SED. Subgroup analyses were performed stratified by gender. Sensitivity analyses were performed by excluding participants who reported symptoms of depression or were identified with chronic disease. A *p* value < 0.05 was considered statistically significant.

## Results

### Description of the study population

Out of 1111 subjects in the SCAPIS pilot study, 795 participants (71.6% of full sample) performed a submaximal cycle ergometer exercise test. Of these subjects, 82 subjects had no/incomplete ISI score, 74 did not complete the accelerometer test, and 15 subjects lacked both assessments. Six hundred twenty-four had complete information on both the ISI questionnaire and the objectively measured physical activity. Subjects with missing information on income-related job, risky drinking, self-reported chronic disease/sleep apnea/depression symptoms, or subjective sleep duration were further excluded from the analyses (*n* = 21). The final cohort included 603 subjects (289 men; mean age 57 ± 4 years; BMI 26.8 ± 4.0 kg/m^2^).

### Non-response analysis

Compared to the final cohort the excluded subjects were slightly older, more overweight, more sedentary and more often current smokers. As expected, self-reported chronic disease, depression symptoms, insomnia, low socioeconomic status, and no university degree as well as no income-related job were more prevalent among these subjects (supplementary table 1). There were no significant differences in VO_2_max or MVPA between the two groups.

### Insomnia vs. non-insomnia

**Fig. 1:**
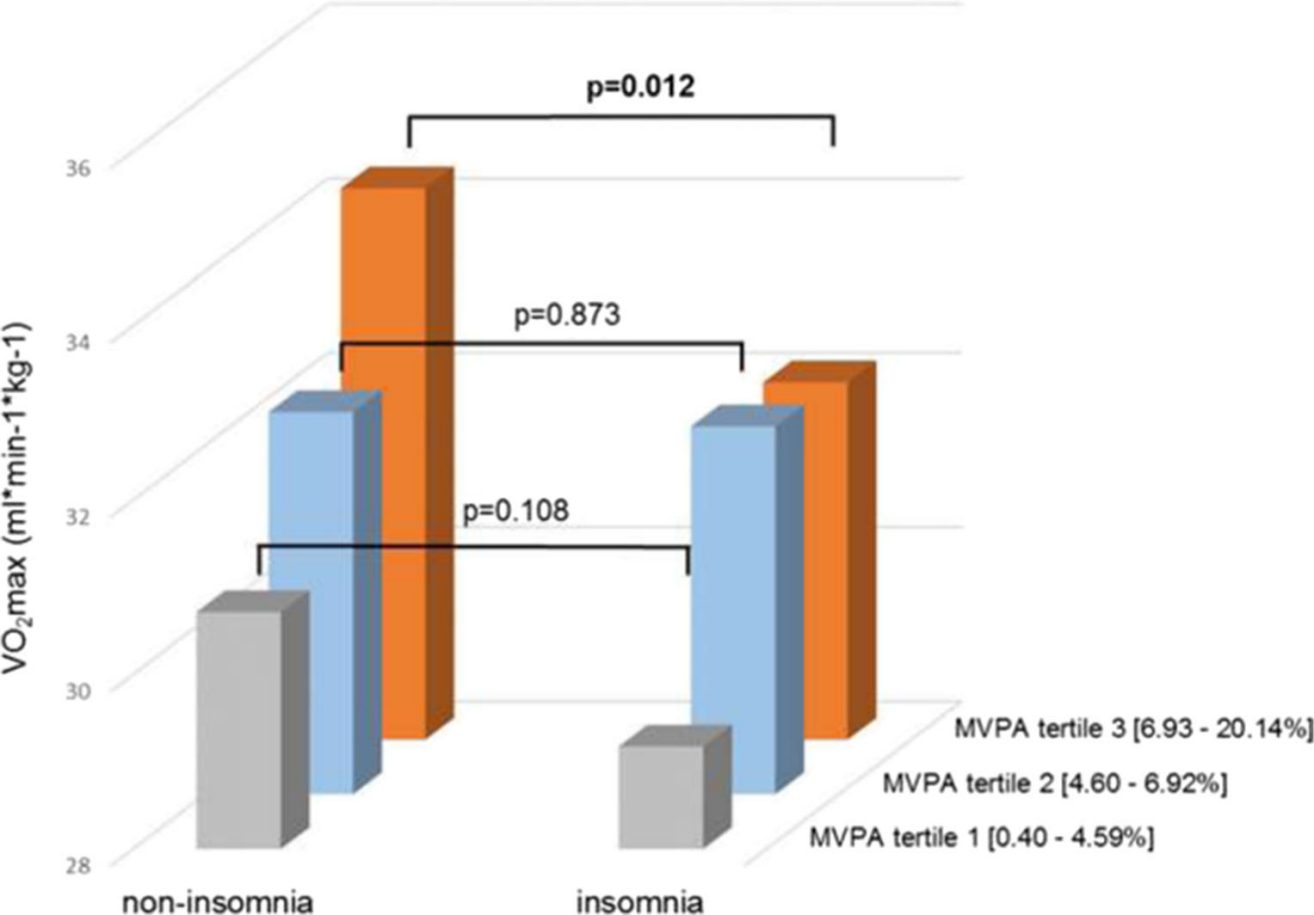
VO_2_max by insomnia and non-insomnia groups across MVPA tertiles

The prevalence of insomnia (ISI ≥ 10) was 31.8% in this middle-aged population. The median ISI score in men and women was 6 [IQR 2 to 10] and 7 [4 to 12], respectively (Mann-Whitney test, *p* < 0.001). The proportion of ISI < 10 in women and men was 64.3% and 72.3%, respectively. As expected, insomnia was more prevalent among women than men (35.7% vs. 27.7%, *p* = 0.035). Compared to the non-insomnia group, there was a higher proportion of low socioeconomic status, no income-related job and depression symptoms in the insomnia group (all *p* < 0.01). There were no significant differences in objectively measured physical activity (MVPA) or SED between the groups. However, VO_2_max was lower in insomnia compared to non-insomnia subjects (31.2 ± 6.3 vs. 32.4 ± 6.5 ml *kg^−1^ *min^−1^, *p* = 0.028, Cohen’s *d* = 0.49, Table 1). Insomnia was consistently associated with lower VO_2_max across the tertiles of MVPA, and VO_2_max showed a significant difference in subjects with the highest MVPA level (Fig. 1).

**Table 1:** Characteristics of the studied cohort by insomnia status (*n* = 603)

	Total	Insomnia severity index < 10 (*n* = 411)	Insomnia severity index ≥ 10 (*n* = 192)	*p* value for comparison
Men (%)	47.9	50.9	41.7	*0.035*
Age (years)	57.2 (4.4)	57.4 (4.4)	56.9 (4.1)	0.148
Body mass index (kg/m^2^)	26.8 (4.0)	26.6 (3.9)	27.2 (4.3)	0.137
Waist circumference (cm)	93.7 (11.4)	93.7 (11.5)	93.7 (11.3)	0.989
Low socioeconomic status (%)	39.8	35.8	48.4	*0.003*
University degree (%)	42.6	45.3	37.0	0.056
No income-related job (%)	17.4	13.6	25.5	*< 0.001*
Smoking (%)				
Never	47.1	48.9	43.2	0.339
Occasional	2.8	3.2	2.1	
Former	40.0	38.9	42.2	
Current	10.1	9.0	12.5	
Risky alcohol consumption (%)	28.9	27.5	31.8	0.280
Subjective sleep duration *<* 6 h (%)	9.0	3.2	21.4	*< 0.001*
Sleep apnea (%)	4.3	3.9	5.2	0.459
Chronic disease (%)	10.9	9.5	14.1	0.094
Depression symptoms (%)	24.0	16.1	41.1	*< 0.001*
Percentage MVPA	5.7 [3.7]	5.5 [3.6]	5.9 [3.6]	0.358
Percentage SED	52.6 (9.8)	52.4 (9.9)	52.8 (9.6)	0.616
VO_2_max (ml *min^−1^ *kg^−1^)	32.0 (6.5)	32.4 (6.5)	31.2 (6.3)	*0.028*

Data show as mean (SD) or median [inter quartile range]. Chronic disease includes stroke, coronary artery disease, diabetes, cancer, and COPD

Significant *p* value presented in italics

*MVPA* moderate to vigorous intensity physical activity, *SED* time spent sedentary

### The association between insomnia and VO_2_max

In a multivariate generalized linear model, insomnia status was not independently associated with VO_2_max (*β* = − 0.56, 95% CI − 1.31–0.20, *p* = 0.148) controlling for age, gender, BMI, waist circumference, SES, university education, income-related job, smoking, risky alcohol consumption, subjective short sleep duration < 6 h, depression symptoms, self-reported chronic disease, sleep apnea, percentage of MVPA, and percentage of SED (Table 2). However, in a gender-stratified multivariate analysis, insomnia was significantly associated with lower VO_2_max in men (*β* = − 1.15, 95% CI − 2.23–− 0.06, *p* = 0.038), but not in women (*β* = − 0.09, 95% CI −1.09–0.92, *p* = 0.866) (Table 3).

**Table 2:** Multivariate generalized linear model for VO_2_max prediction in the full sample (*n* = 603)

	*β*-coefficients (95% CI)	*p* value
Men vs. women	*7.47 (6.64–8.30)*	*< 0.001*
Age (years)	*− 0.32 (− 0.40–− 0.25)*	*< 0.001*
BMI (kg/m^2^)	*− 0.50 (− 0.66–− 0.33)*	*< 0.001*
Waist circumference (cm)	*− 0.16 (− 0.22–− 0.10)*	*< 0.001*
Low vs. high socioeconomic status	*− 1.30 (− 2.02–− 0.59)*	*< 0.001*
With vs. without university education	*0.97 (0.27–1.67)*	*0.007*
Without vs. with income-related job	0.68 (*−* 0.24–1.60)	0.146
Smoking		
Occasional vs. never	1.15 (*−* 0.84–3.13)	0.257
Former vs. never	*−* 0.12 (*−* 0.83–0.60)	0.753
Current vs. never	0.02 (*−* 1.14–1.18)	0.978
Risky vs. not risky alcohol consumption	0.42 (*−* 0.32–1.16)	0.265
Insomnia vs. non-insomnia	*−* 0.56 (*−* 1.31–0.20)	0.148
Subjective sleep duration *<* 6 h vs. ≥ 6 h	0.55 (*−* 0.66–1.75)	0.377
Sleep apnea vs. no apnea	1.51 (*−* 0.13–3.15)	0.071
Chronic disease vs. no disease	*−* 0.45 (*−* 1.49–0.58)	0.390
Depression symptoms vs. no symptoms	0.43 (*−* 0.37–1.23)	0.293
Percentage (%) MVPA	*0.24 (0.12–0.37)*	*< 0.001*
Percentage (%) SED	0.01 (*−* 0.03–0.05)	0.541

Chronic disease includes stroke, coronary artery disease, diabetes, cancer, and COPD

Significant *p* value presented in italics

*MVPA* moderate to vigorous intensity physical activity, *SED* time spent sedentary

**Table 3:** Multivariate generalized linear model for VO2max prediction stratified by gender

	Men (*n* = 289)	
	*β*-coefficient (95% CI)	*p* value
Insomnia vs. non-insomnia	*− 1.15 (− 2.23–− 0.06)*	*0.038*
	Women (*n* = 314)	
	*β*-coefficient (95% CI)	*p* value
Insomnia vs. non-insomnia	*−* 0.09 (*−* 1.09–0.92)	0.866

Adjusting for age, body mass index, waist circumference, socioeconomic status, university education, income-related job, smoking, risky drinking, chronic disease (including stroke, coronary artery disease, diabetes, cancer, and COPD), sleep apnea, depression symptoms, sleep duration *<* 6 h, percentage of moderate to vigorous intensity physical activity, and percentage of time spent sedentary

Significant *p* value presented in italics

### Sensitivity analyses

In a repeated sensitivity analysis excluding 66 subjects with self-reported chronic disease, the result between insomnia and VO_2_max remained unchanged (supplementary tables 2 and 3). Similarly, when excluding subjects with self-reported symptoms of depression (*n* = 145), the association between insomnia and lower VO_2_max in men was preserved (supplementary tables 4 and 5).

## Discussion

In this middle-aged gender-balanced cohort, insomnia detected by ISI score ≥ 10 was associated with lower CRF, particularly in men. This association was independent of SES, body composition, comorbidity, and lifestyle including MVPA. To the best of our knowledge, this is the first study using a valid insomnia diagnosis instrument rather than insomnia symptoms to address the link between CRF and insomnia. It is argued that in relation to, and possibly in addition to, unhealthy lifestyle (e.g., physical inactivity) low CRF may constitute an important link to increased cardiometabolic risk in insomnia.

Both physical activity and CRF provide strong prognostic ability for future cardiovascular disease. CRF is moderately related to the intense aspect of habitual physical activity and has been observed to be a stronger predictor of cardiovascular events and mortality than physical activity [24, 25]. Although the insomnia–low physical activity association has been well studied, the relationship between insomnia and CRF remains less explored. Regular moderate to vigorous exercise may increase sleep time and improve sleep consolidation through mechanisms such as altered body core temperature and increased brain-derived neurotropic factor activity [26]. Physical exercise induces an acute rise, but over longer term reduction, of growth hormone and testosterone levels. The magnitude of this response is influenced by factors such as previous exercise levels, age, and nutritional status [27, 28]. The role of an increase in growth hormone level for the subsequent night sleep is inconclusive and it remains uncertain whether physical exercise can alter the circadian fluctuations in hormone levels including testosterone and cortisol [29]. Nevertheless, elevated sympathetic activity, increased cortisol levels, and inflammation caused by insomnia may lead to lowered alertness, mood disorders, and higher rate of perceived exertion which may limit physical performance by reduced effort [26].

Indeed, a lower CRF in insomnia, as compared with non-insomnia subjects, was observed in the current study indicating that insomnia as a chronic disease may influence the complex biological processes involving the integrative coordination of multiple physiological systems for O_2_ metabolism and transport. In gender-specific analyses, an inverse association between insomnia and CRF was found in men but not in women. The exact explanation of this gender-specific effect is unclear. However, it is known that subjects who participated in the fitness test are generally healthier than those not participating, due to the exclusion criteria applied for the exercise test. In the current study, the prevalence of insomnia was lower in the included women compared to women excluded from the analyses. This may have reduced the power to detect a significant association between insomnia and CRF in women.

The ISI used in the current study provides a more comprehensive assessment of nighttime and daytime components of insomnia compared with information on insomnia symptoms alone. An ISI cutoff of 10 has been shown to detect insomnia cases in a community sample with 86% sensitivity and 88% specificity [16]. The prevalence of insomnia in the current study is comparable to that reported in a Swedish population–based survey [30]. In line with other epidemiological studies, we found that female gender, low social economy status, no income-related job, and depression symptoms were overrepresented in the insomnia group [1]. In our study, more than 20% of the subjects with insomnia reported a sleep duration of less than 6 h similar to another population-based study [31]. Short sleep duration has been shown to independently predict cardiovascular morbidity [5, 32], and it was argued that studies on insomnia and cardiovascular disease should take into account the impact of short habitual sleep time [33]. Therefore, we included subjective short sleep duration in our multivariate analysis and an independent association between insomnia and CRF remained, in men.

The observed difference (1.15 ml* kg^−1^ *min^−1^) in CRF between the male insomnia and non-insomnia groups is modest but of clinical relevance. The age-related decline in VO_2_max has been reported to be 0.26 ml *kg^−1^ *min^−^1 per year in men [34]. A meta-analysis of 102,980 healthy men and women indicated that each 3.5 ml *kg^−1^ *min^−1^ increase in CRF is associated with a 13% lower risk of all-cause mortality and 15% lower risk of cardiovascular morbidity [11]. It has also been shown that men with CRF corresponding to the lowest quintile have a substantially greater risk for all-cause mortality as compared with men with moderate or high levels of CRF [35, 36]. A recent statement from the American Heart Association has emphasized the importance of CRF assessment in clinical practice, either by measurement or estimation, in order to optimize the prevention and treatment of cardiovascular disease [10]. In the current study, a submaximal cycle ergometer test was applied instead of a maximal protocol with ventilatory gas analyses in order to increase the feasibility of the protocol in the population-based setting. A newly developed equation with improved validity was used for VO_2_max estimation [19]. It is believed that this approach enables a more representative cohort and increases the generalizability of the result.

Despite the gender-balanced design, inclusion of submaximal exercise testing and objectively measured physical activity, our study also comes with limitations. Despite the fact that ISI is a valid diagnostic instrument for insomnia, the intensity and duration of the disease are not addressed. Although adequate, the use of an ISI cutoff of 10 could have led to an identification of more subjects with mild insomnia in the current study. This could, in part, have attributed to the small difference in VO_2_max observed in the insomnia and non-insomnia groups. Depression symptoms and sleep apnea were assessed by single questions rather than by validated questionnaires. The cross-sectional design of the current study does not allow any conclusions on causality for the relationship between insomnia and CRF. It has recently been shown that a decline in CRF increases the odds of incident sleep complaints [37]. Whether the observed difference in VO_2_max between insomnia and non-insomnia men is due to altered physiological adaptation to physical activity needs to be further studied. Finally, the age range of the cohort may limit the generalizability of the results. Prospective studies with larger age range and detailed mapping of insomnia subtype are warranted to further elucidate the relationship between insomnia and CRF.

## Conclusion

In this urban mid-aged Swedish population, we demonstrated that insomnia is associated with lower CRF in men, independent of body composition, living conditions, comorbidity, and lifestyle, including objectively assessed physical activity. CRF represents the body’s adaptability to physical exertion which may be limited by insomnia. In light of the strong association between low CRF and increased cardiometabolic disease risk, our results provide a possible link of insomnia to cardiovascular morbidity and mortality.

## Electronic supplementary material

ESM 1 (DOCX 25.5 kb)
