# Supplementary material for: Insomnia and cardiorespiratory fitness in a middle-aged population: the SCAPIS pilot study
Source: Sleep Breath. 2018 Dec 13;23(1):319–26. doi: 10.1007/s11325-018-1765-9 (PMC6418054; doi:10.1007/s11325-018-1765-9)
Supplement: Supplementary file 1 — (DOCX 25.5 kb) [file 11325_2018_1765_MOESM1_ESM.docx]

**Insomnia and cardiorespiratory fitness in a middle-aged population: the SCAPIS pilot study**

Ding Zou^1*^, Heini Wennman^2*^, Örjan Ekblom^3^, Ludger Grote^1^, Daniel Arvidsson^4^, Anders Blomberg^5^, Kjell Torén^6^, Göran Bergström^7,8^, Mats Börjesson^4,8^, and Jan Hedner^1^

^1^ Center for Sleep and Vigilance Disorders, Department of Internal Medicine and Clinical Nutrition, Sahlgrenska Academy, University of Gothenburg, Gothenburg, Sweden

^2^ Department of Public Health Solutions, National Institute for Health and Welfare, Helsinki, Finland

^3^ Åstrand Laboratory of Work Physiology, The Swedish School of Sport and Health Sciences, Stockholm, Sweden

^4^ Center for Health and Performance, Department of Food and Nutrition, and Sport Science, Institute of Neuroscience and Physiology, University of Gothenburg, Gothenburg, Sweden

^5^ Department of Public Health and Clinical Medicine, Division of Medicine/Respiratory Medicine, Umeå University, Umeå, Sweden

^6^ Section of Occupational and Environmental Medicine, Sahlgrenska Academy, University of Gothenburg, Gothenburg, Sweden

^7^ Department of Molecular and Clinical Medicine, Institute of Medicine, Sahlgrenska Academy,

University of Gothenburg, Gothenburg, Sweden

^8^ Sahlgrenska University Hospital, Gothenburg, Sweden

*These authors contributed equally to this work

Supplementary table 1. Comparison between selected and non-selected subjects of the total SCAPIS pilot cohort (n=1111).

|  | Selected n=603 | | | Not selected | | |
| --- | --- | --- | --- | --- | --- | --- |
|  | All | Men | Women | All | Men | Women |
| Men (%) | 47.9 |  |  | 52.4 |  |  |
| Age (years) | **57.2 (4.4)*** | **57.4 (4.4)¤** | **57.1 (4.3)#** | **58.2 (4.4)*** | **58.2 (4.5)¤** | **58.2 (4.3)#** |
| Body mass index (kg/m^2^) | **26.8 (4.0)*** | **27.1 (3.4)¤** | 26.5 (4.5) | **27.9 (5.0)*** | **28.4 (4.4)¤** | 27.3 (5.4) |
| Waist circumference (cm) | **93.7 (11.4)*** | **98.3 (9.1)¤** | 89.4 (11.7) | **97.6 (14.0)*** | **103.1 (11.8)¤** | 91.6 (13.8) |
| Low socioeconomic status (%) | **39.8*** | **41.2¤** | **38.5#** | **61.4*** | **60.2¤** | **62.8#** |
| University degree (%) | **42.6*** | 37.0 | **47.8#** | **30.2*** | 29.5 | **30.9#** |
| No income related job (%) | **17.4*** | **19.0¤** | **15.9#** | **35.3*** | **33.5¤** | **37.4#** |
| Smoking (%) |  |  |  |  |  |  |
| Never | **47.1*** | **48.4¤** | **45.9#** | **38.2*** | **33.0¤** | **44.1#** |
| Occasional | **2.8*** | **1.4¤** | **4.1#** | **4.0*** | **3.8¤** | **4.2#** |
| Former | **40.0*** | **40.5¤** | **39.5#** | **37.5*** | **42.8¤** | **31.5#** |
| Current | **10.1*** | **9.7¤** | **10.5#** | **20.3*** | **20.5¤** | **20.2#** |
| Risky alcohol consumption (%) | 28.9 | 36.7 | 21.7 | 27.3 | 30.9 | 23.1 |
| Insomnia  (ISI ≥10) (%) | **31.8*** | 27.7 | **35.7#** | **39.3*** | 30.4 | **48.8#** |
| Subjective sleep duration <6 hours (%) | **9.0*** | 8.7 | **9.2#** | **15.6*** | 13.1 | **18.4#** |
| Sleep apnea (%) | 4.3 | 5.9 | 2.9 | 6.9 | 8.5 | 5.2 |
| Chronic disease (%) | **10.9*** | **8.7¤** | **13.1#** | **25.2*** | **27.0¤** | **23.1#** |
| Depression symptoms (%) | **24.0*** | **16.3¤** | 31.2 | **31.2*** | **25.9¤** | 37.1 |
| Percentage MVPA (%) | 6.0 (2.9) | 6.1 (3.1) | **6.0 (2.7)#** | 5.7 (3.7) | 6.4 (4.1) | **5.1 (3.2)#** |
| Percentage SED (%) | **52.6 (9.8)*** | 55.1 (9.9) | **50.2 (9.1)#** | **54.0 (10.6)*** | 55.6 (10.9) | **52.4 (10.0)#** |
| VO_2_max  (ml*min^-1^*kg^-1^) | 32.0 (6.5) | **35.0 (5.5)¤** | 29.3 (6.0) | 31.4 (6.5) | **33.4 (5.7)¤** | 29.1 (6.5) |

ISI= insomnia severity index; MVPA= moderate to vigorous physical activity; SED= time spent sedentary; Chronic disease include stroke, coronary artery disease, diabetes, cancer, and COPD.

*Significant difference between selected and not selected group.

#Significant difference between selected women and not selected women.

¤Significant difference between selected men and not selected men.

Supplementary table 2. Multivariate generalized linear model for VO_2_max prediction in the cohort excluding subjects with chronic disease (n=537).

|  | β-coefficients (95% CI) | p-value |
| --- | --- | --- |
| Men vs. women | **7.40 (6.50 – 8.30)** | **<0.001** |
| Age (years) | **-0.32 (-0.41 – -0.24)** | **<0.001** |
| BMI (kg/m^2^) | **-0.51 (-0.69 – -0.34)** | **<0.001** |
| Waist circumference (cm) | **-0.16 (-0.23 – -0.09)** | **<0.001** |
| Low vs. high socioeconomic status | **-1.37 (-2.13 – -0.60)** | **<0.001** |
| With vs. without university education | **0.98 (0.22 – 1.74)** | **0.011** |
| Without vs. with income related job | 0.59 (-0.42 – 1.60) | 0.250 |
| Smoking |  |  |
| Occasional vs. never | 1.14 (-0.93 – 3.21) | 0.280 |
| Former vs. never | -0.07 (-0.83 – 0.70) | 0.867 |
| Current vs. never | -0.10 (-1.31 – 1.12) | 0.877 |
| Risky vs. not risky alcohol consumption | 0.40 (-0.39 – 1.19) | 0.322 |
| Insomnia vs. non-insomnia | -0.46 (-1.28 – 0.35) | 0.263 |
| Subjective sleep duration <6 hours vs. ≥6 hours | 0.45 (-0.89 – 1.79) | 0.509 |
| Sleep apnea vs. no apnea | 1.61 (-0.15 – 3.37) | 0.072 |
| Depression symptoms vs. no symptoms | 0.45 (-0.42 – 1.33) | 0.307 |
| Percentage (%) MVPA | **0.24 (0.10 – 0.37)** | **0.001** |
| Percentage (%) SED | 0.01 (-0.03 – 0.06) | 0.548 |

MVPA = moderate to vigorous intensity physical activity; SED = time spent sedentary. Chronic disease include stroke, coronary artery disease, diabetes, cancer, and COPD.

Supplementary table 3. Multivariate generalized linear model for VO_2_max prediction excluding subjects with chronic disease stratified by gender.

|  | Men (n=264) | |
| --- | --- | --- |
|  | β-coefficient (95% CI) | p-value |
| Insomnia vs. Non-insomnia | **-1.20 (-2.35 – -0.05)** | **0.041** |
|  | Women (n=273) | |
|  | β-coefficient (95% CI) | p-value |
| Insomnia vs. Non-insomnia | 0.25 (-0.84 – 1.34) | 0.652 |

Adjusting for age, body mass index, waist circumference, socioeconomic status, university education, income related job, smoking, risky drinking, sleep apnea, depression symptoms, sleep duration <6 hours, percentage of moderate to vigorous intensity physical activity, and percentage of time spent sedentary.

Supplementary table 4. Multivariate generalized linear model for VO_2_max prediction in the cohort excluding subjects with symptoms of depression (n=458).

|  | β-coefficients (95% CI) | p-value |
| --- | --- | --- |
| Men vs. women | **7.46 (6.51 – 8.41)** | **<0.001** |
| Age (years) | **-0.34 (-0.42 – -0.25)** | **<0.001** |
| BMI (kg/m^2^) | **-0.43 (-0.63 – -0.24)** | **<0.001** |
| Waist circumference (cm) | **-0.19 (-0.26 – -0.12)** | **<0.001** |
| Low vs. high socioeconomic status | **-1.42 (-2.25 – -0.59)** | **0.001** |
| With vs. without university education | **0.86 (0.06 – 1.67)** | **0.036** |
| Without vs. with income related job | 0.73 (-0.41 – 1.86) | 0.209 |
| Smoking |  |  |
| Occasional vs. never | 1.81 (-0.54 – 4.15) | 0.130 |
| Former vs. never | -0.03 (-0.84 – 0.79) | 0.945 |
| Current vs. never | -0.37 (-1.76 – 1.03) | 0.605 |
| Risky vs. not risky alcohol consumption | 0.18 (-0.66 – 1.02) | 0.670 |
| Insomnia vs. non-insomnia | -0.57 (-1.47 – 0.33) | 0.213 |
| Subjective sleep duration <6 hours vs. ≥6 hours | 0.88 (-0.74 – 2.49) | 0.288 |
| Sleep apnea vs. no apnea | 1.81 (-0.07 – 3.69) | 0.059 |
| Chronic disease vs. no disease | -0.41 (-1.68 – 0.85) | 0.521 |
| Percentage (%) MVPA | **0.21 (0.07 – 0.35)** | **0.004** |
| Percentage (%) SED | 0.02 (-0.02 – 0.07) | 0.309 |

MVPA = moderate to vigorous intensity physical activity; SED = time spent sedentary. Chronic disease include stroke, coronary artery disease, diabetes, cancer, and COPD.

Supplementary table 5. Multivariate generalized linear model for VO_2_max prediction excluding subjects with symptoms of depression, stratified by gender.

|  | Men (n=242) | |
| --- | --- | --- |
|  | β-coefficient (95% CI) | p-value |
| Insomnia vs. Non-insomnia | **-1.38 (-2.62 – -0.13)** | **0.031** |
|  | Women (n=314) | |
|  | β-coefficient (95% CI) | p-value |
| Insomnia vs. Non-insomnia | -0.08 (-1.16 – 1.32) | 0.898 |

Adjusting for age, body mass index, waist circumference, socioeconomic status, university education, income related job, smoking, risky drinking, chronic disease (including stroke, coronary artery disease, diabetes, cancer, and COPD), sleep apnea, sleep duration <6 hours, percentage of moderate to vigorous intensity physical activity, and percentage of time spent sedentary.
